# Rap1 is indispensable for TRF2 function in etoposide-induced DNA damage response in gastric cancer cell line

**DOI:** 10.1038/oncsis.2015.1

**Published:** 2015-03-30

**Authors:** X Li, W Liu, H Wang, L Yang, Y Li, H Wen, H Ning, J Wang, L Zhang, J Li, D Fan

**Affiliations:** 1 Department of Neurology, Second Affiliated Hospital of Zhengzhou University, Zhengzhou, China; 2 Department V of Digestive Diseases, First Affiliated Hospital of Zhengzhou University, Zhengzhou, China; 3 Department II of Digestive Diseases, First Affiliated Hospital of Zhengzhou University, Zhengzhou, China; 4 State Key Laboratory of Cancer Biology and Xijing Hospital of Digestive Diseases, The Fourth Military Medical University, Xi'an, China

## Abstract

The telomeric protein TRF2, involving in telomeric and extratelomeric DNA damage response, has been previously reported to facilitate multidrug resistance (MDR) in gastric cancer cells by interfering ATM-dependent DNA damage response induced by anticancer drugs. Rap1 is the TRF2-interacting protein in the shelterin complex. Complex formation between Rap1 and TRF2 is essential for their function in telomere and end protection. Here we focus on the effects of Rap1 on TRF2 function in DNA damage response induced by anticancer drugs. Both Rap1 and TRF2 expression were upregulated in SGC7901 and its MDR variant SGC7901/VCR after etoposide treatment, which was more marked in SGC7901/VCR than in SGC7901. Rap1 silencing by siRNA in SGC7901/VCR partially reversed the etoposide resistance. And Rap1 silencing partially reversed the TRF2-mediated resistance to etoposide in SGC7901. Rap1 silencing did not affect the TRF2 upregulation induced by etoposide, but eliminated the inhibition effect of TRF2 on ATM expression and ATM phosphorylation at serine 1981 (ATM pS1981). Furthermore, phosphorylation of ATM targets, including γH2AX and serine 15 (S15) on p53, were increased in Rap1 silencing cells in response to etoposide. Thus, we confirm that Rap1, interacting with TRF2 in the shelterin complex, also has an important role in TRF2-mediated DNA damage response in gastric cancer cells treated by etoposide.

## Introduction

The telomeric protein TRF2 has been evaluated to be a general DNA-repair factor relevant with both telomeric and extratelomeric DNA damage response.^1, 2^ TRF2 accumulates at double-strand break sites in the first few seconds after irradiation as an early response to DNA damage.^3^ Phosphorylated by ataxia-telangiectasia mutated (ATM), TRF2 in turn inhibits activation of ATM-dependent DNA damage response.^4, 5^ TRF2 interacts with double-strand break repair proteins and involves in homologous recombination repair of nontelomeric double-strand breaks.^6^ In our previous study, it showed that TRF2 promoted multidrug resistance (MDR) of gastric cancer cells by interfering DNA damage response induced by anticancer drugs.^7^

Rap1 is the TRF2-interacting protein in the shelterin complex. Complex formation between Rap1 and TRF2 is essential for their function in telomere and end protection. Rap1 relies on TRF2 for stable expression.^8^ Human Rap1 (hRap1) is recruited to telomeres through interaction with TRF2.^9^ hRap1 alters the affinity of hTRF2 and its binding preference on telomeric DNA. The TRF2-Rap1 complex possesses higher ability to remodel telomeric DNA than either component alone.^10^ Rap1 could be potentially involved in the function of TRF2 in DNA damage response,^11^ whereas it remains unknown whether TRF2 requires Rap1 for its function in DNA damage response induced by anticancer drugs.

In this paper, we aimed to investigate changes of Rap1 and TRF2 expression in human gastric cells (SGC7901 and SGC7901/VCR) treated by etoposide, summarize effects of Rap1 silencing on the TRF2 expression and DNA damage response and identify the phosphorylation of ATM targets. Subsequently, the association between Rap1 and TRF2 was discussed to further the relevant study on the possible mechanism of multidrug resistance and develop effective methods against the multidrug resistance.

## Results

### TRF2 and Rap1 expression induced by etoposide treatment in gastric cancer cells

**Figure 1:**
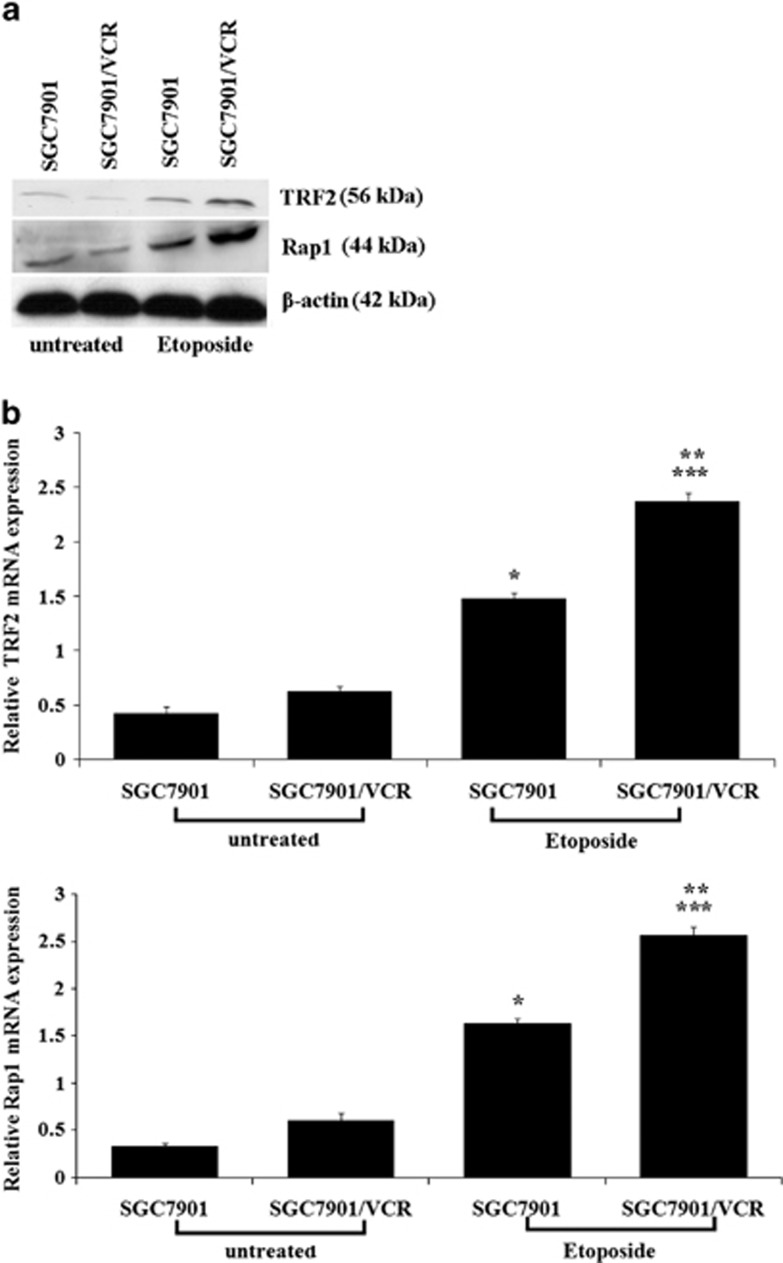
TRF2 and Rap1 upregulation induced by etoposide in gastric cancer cells. (**a**) Protein expression levels of TRF2 and Rap1 detected by western blot in SGC7901 and SGC7901/VCR after treated with 20 μg/ml of etoposide for 6 h. (**b**) Relative mRNA expression levels of TRF2 and Rap1 detected by real-time PCR analysis in SGC7901 and SGC7901/VCR after treated with 20 μg/ml of etoposide for 6 h. *P<0.0001 vs untreated-SGC7901; **P<0.0001 vs untreated-SGC7901/VCR; ***P<0.0001 vs etoposide-SGC7901.

In previous work, we reported that etoposide or adriamycin (ADR) treatment upregulated TRF2 expression in gastric cancer cells, which occurred at early stage of DNA damage response and showed dose-dependency. On the basis of the significance of the Rap1-TRF2 complex,^10, 11^ we detected the presence of co-upregulated TRF2 and Rap1 expression in gastric cancer cells after etoposide treatment. Treated with 20 μg/ml etoposide for 6 h,^7^ the mRNA and protein levels of TRF2 and Rap1 in SGC7901 and SGC7901/VCR cells were determined by western blot and real-time PCR. As shown in Figure 1, both TRF2 and Rap1 expression were upregulated, which were higher in SGC7901/VCR than in SGC7901.

### Involvement of Rap1 in TRF2-mediated etoposide resistance in gastric cancer cells

**Figure 2:**
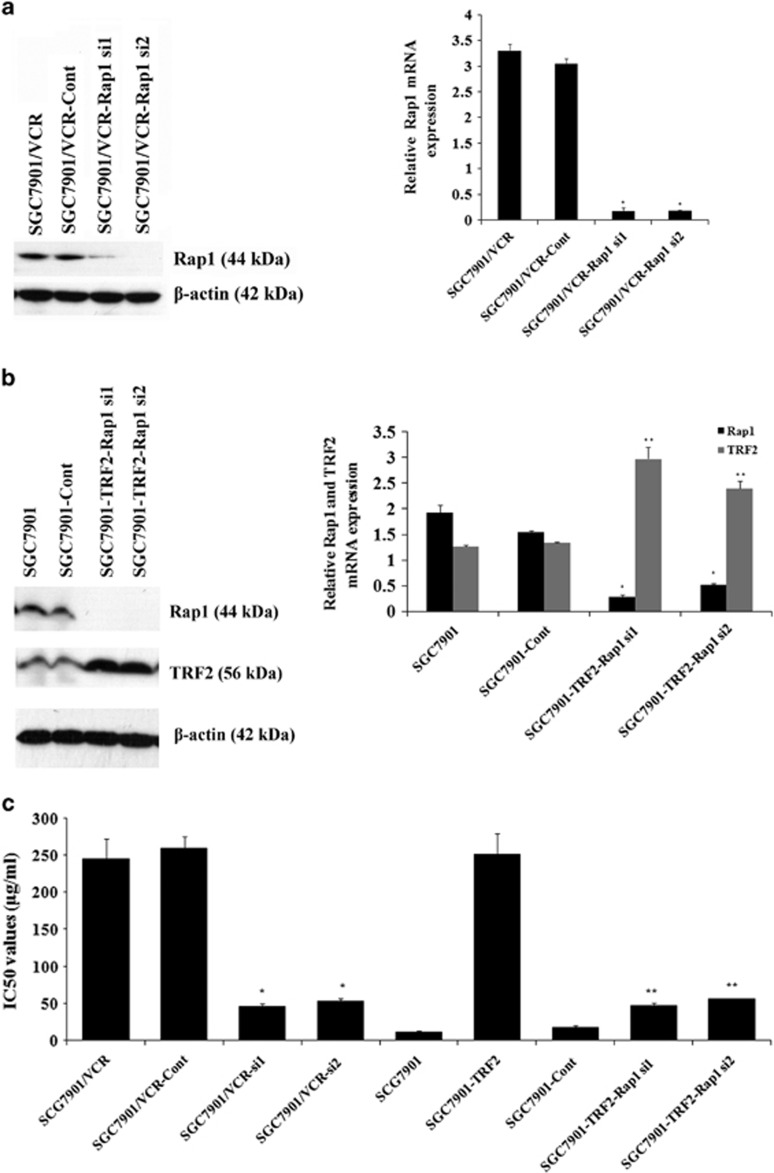
Rap1 is involved in TRF2-mediated etoposide resistance in gastric cancer cells. (**a**) Rap1 siRNA vectors were transfected into SGC7901/VCR, respectively. Rap1 expression after treated with 20 μg/ml of etoposide for 6 h was detected by western blot and real-time PCR analysis. *P<0.0001 vs SGC7901/VCR and SGC7901/VCR-Cont. (**b**) Rap1 siRNA vector and TRF2 eukaryotic expression vector were cotransfected into SGC7901. TRF2 and Rap1 expression after treated with 20 μg/ml of etoposide for 6 h were detected by western blot and real-time PCR analysis. *P<0.0001 and **P<0.0001 vs SGC7901 and SGC7901-Cont. (**c**) IC_50_ values (μg/ml) of gastric cancer cells for etoposide. The sensitivity of gastric cancer cells to etoposide was evaluated using MTT assay as described in Materials and Methods section. The concentration of etoposide that caused a 50% reduction in number of colonies (IC_50_) was calculated. *P<0.01 vs SGC7901/VCR and SGC7901/VCR-Cont; **P<0.01 vs SGC7901–TRF2 and SGC7901-Cont.

Two Rap1 siRNA eukaryotic expression vectors were constructed and transiently transfected into SGC7901/VCR, respectively, to identify whether Rap1 participates in TRF2-mediated resistance to etoposide in gastric cancer cells. As shown in Figure 2a, western blot and real-time PCR analysis revealed that Rap1 expression was significantly suppressed after transfection, comparing with those observed in parental cells and control cells. Sensitivity of gastric cancer cells to etoposide was evaluated by 3-(4,5-dimethylthiazol-2-yl)-2,5-diphenyl-tetrazolium bromide (MTT) assay. Results showed that the inhibited Rap1 expression significantly enhanced the sensitivity of SGC7901/VCR to etoposide, indicating that Rap1 might be involved in etoposide resistance of gastric cancer cells (Figure 2c).

Previous research has shown that transient transfection of TRF2 eukaryotic expression vector into SGC7901 cells induced TRF2 overexpression and facilitated emergence of MDR phenotype.^7^ On the basis of this, cotransfection of Rap1 siRNA vector and TRF2 eukaryotic expression vector was performed into SGC7901 cells transiently, resulting in TRF2 overexpression, low Rap1 expression and increased sensitivity to etoposide as compared with SGC7901–TRF2 cells (Figures 2b and c). Taken together, these results demonstrated that Rap1 inhibition in gastric cancer cells could reverse the TRF2-mediated resistance to etoposide.

### Significance of Rap1 to the function of TRF2 in etoposide-induced DNA damage response

**Figure 3:**
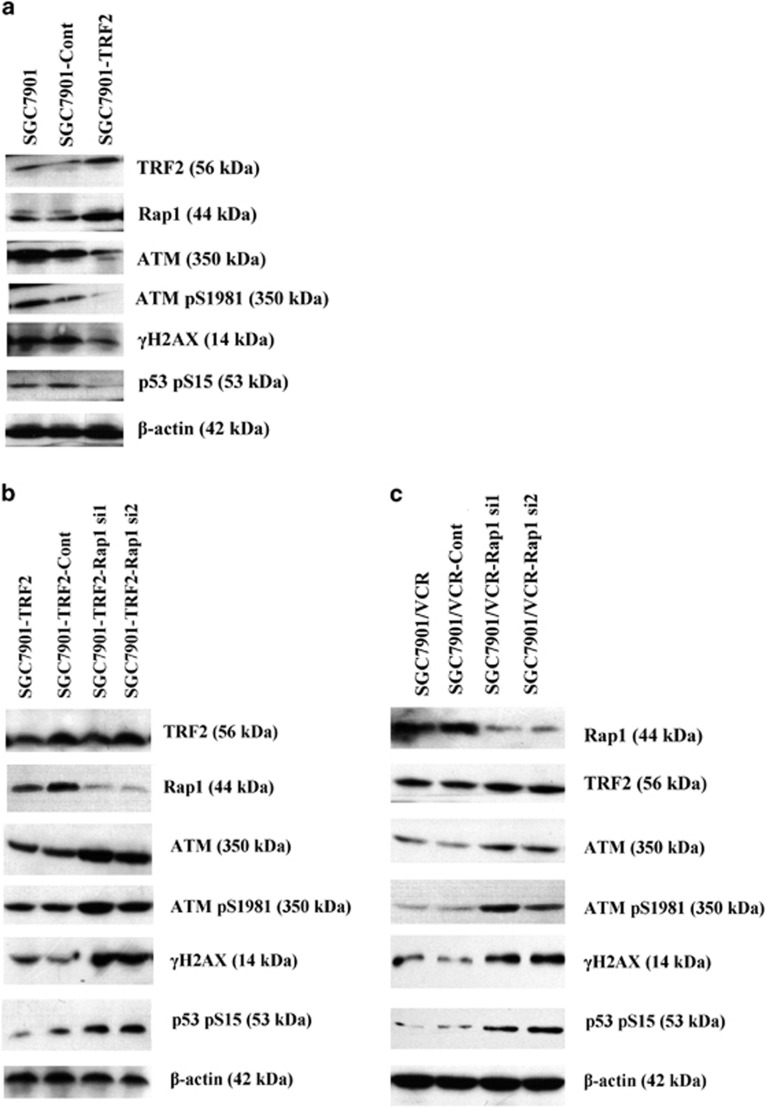
Downregulation of Rap1 eliminated the inhibition effects of TRF2 on etoposide-induced ATM activation. (**a**) TRF2 eukaryotic expression vector was transfected into SGC7901. Expression of ATM, ATM pS1981, γH2AX and p53 pS15 after treated with 20 μg/ml of etoposide for 6 h were detected by western blot. (**b**) Rap1 siRNA vector and TRF2 eukaryotic expression vector were cotransfected into SGC7901. Expression of ATM, ATM pS1981, γH2AX and p53 pS15 after treated with 20 μg/ml of etoposide for 6 h were detected by western blot. (**c**) Rap1 siRNA vector was transfected into SGC7901/VCR. Expression of TRF2, Rap1, ATM, ATM pS1981, γH2AX and p53 pS15 after treated with 20 μg/ml of etoposide for 6 h were detected by western blot.

On the basis of previous investigation, it has been validated that TRF2 promotes MDR of gastric cancer cells by interfering the activation of ATM-dependent DNA damage response.^7^ To further investigate how Rap1 affect TRF2-mediated resistance to etoposide in gastric cancer cells, overexpression of TRF2 in SGC7901 was firstly obtained after transfected with TRF2 eukaryotic expression vector. SGC7901–TRF2 cells showed significant reduction of etoposide-induced ATM expression and ATM phosphorylation at serine 1981 (ATM pS1981). Correspondingly, the phosphorylation of ATM targets, including γH2AX and serine 15 (S15) on p53, were also reduced (Figure 3a). Further inhibition Rap1 by siRNA in SGC7901–TRF2 cells reversed the inhibition effect of TRF2 on ATM expression and activation of ATM-dependent signaling cascade (Figure 3b). Subsequently, etoposide was applied to SGC7901/VCR and its Rap1 siRNA transfectants. Results indicated that Rap1 silencing did not affect the TRF2 upregulation induced by etoposide, but enhance ATM expression and ATM-dependent signaling cascade activation (Figure 3c), suggesting that Rap1 was indispensable for the function of TRF2 in etoposide-induced DNA damage response.

## Discussion

In this paper, it is demonstrated that Rap1 is involved in TRF2-mediated resistance to etoposide in gastric cancer cells. Rap1 silencing did not affect the TRF2 upregulation induced by etoposide, but eliminated the inhibition effect of TRF2 on ATM signals. In combination with our previous work, these results provided powerful evidence that Rap1 is indispensable for the essential functions of TRF2 in DNA damage response, to promote MDR of gastric cancer cells.

Recently, expression changes of the six shelterin components in response to topoisomerase II inhibitors have been reported.^12^ The mRNA levels of Rap1 were upregulated in human fetal lung fibroblasts and human osteosarcoma cells with doxorubicin and etoposide treatment.^13^

Here we advanced that both Rap1 and TRF2 expression in gastric cancer cells were upregulated after etoposide treatment. Furthermore, the Rap1 and TRF2 upregulation induced by etoposide were more marked in multidrug-resistant cells, suggesting the function of Rap1 and TRF2 in tumor MDR.

To study effects of Rap1 on TRF2-mediated resistance to etoposide in gastric cancer cells, we first inhibited Rap1 expression in SGC7901/VCR by RNA interference. Transient transfection was used to eliminate the effect of Rap1 and TRF2 on telomere length and genomic stability.^7^ The inhibited Rap1 expression significantly resulted in the enhanced sensitivity of resistant cancer cells to etoposide. We further cotransfected SGC7901 cells with Rap1 siRNA vector and TRF2 eukaryotic expression vector transiently and found that etoposide resistance of SGC7901–TRF2 cells induced by TRF2 overexpression could be reversed by Rap1 inhibition. Overall, these experimental data indicated Rap1 is closely connected with TRF2-mediated etoposide resistance of gastric cancer cells.

TRF2 was recently reported to be an early component of the DNA-repair response system. In DNA damage caused by irradiation, TRF2 is rapidly phosphorylated and localizes to the damage sites.^14^ TRF2 directly interacts with ATM in the S1981surrounding region *in vitro* and inhibits ATM activation. It is been revealed that TRF2 inhibits cell cycle arrest and other ATM-dependent readouts of the DNA damage response resulted from the ionizing radiation.^15^ Our previous studies also showed that inhibition of ATM signals might be the potential mechanism of TRF2-mediated MDR in gastric cancer cells. And in this paper, Rap1 silencing did not affect the TRF2 upregulation induced by etoposide, but eliminated the inhibiting effect of TRF2 on activation of ATM-dependent kinase cascade. Consequently, Rap1 might be indispensable for TRF2 function in the etoposide-induced DNA damage response.

In summary, Rap1 is critical to the TRF2-mediated etoposide resistance in gastric cancer cells. The underlying mechanism might be associated with the regulated response of TRF2 to DNA damage response induced by anticancer drugs. As Rap1 has been described as an adapter protein mediating diverse protein–protein and protein–DNA interactions,^16^ further study of the interaction between Rap1 and TRF2 might be conducive to understanding the intrinsic property of MDR and developing possible strategies to overcome MDR.

## Materials and methods

### Cells and reagents

Human gastric cancer cell line SGC7901 and its multidrug-resistant cell variant SGC7901/VCR were obtained from State Key Laboratory of Cancer Biology (Xi'an, China).^17^ All the cell lines were maintained in RPMI1640 medium (Invitrogen, Carlsbad, CA, USA) supplemented with 10% (v/v) heat-inactivated fetal calf serum and antibiotics at 37 °C with 5% CO_2_ in a humidified incubator (Forma Scientific, Marietta, OH, USA).

### Western blot analysis

Cells were collected, and total cellular proteins were prepared with lysis buffer (50 mm Tris (pH 8.0), 150 mM NaCl, 0.02% NaN_3_, 0.1% SDS, 100 μg/ml PMSF, 1 μg/ml Aprotinin, 1%NP-40, 0.5% sodium orthovanadate). Proteins were resolved by SDS–PAGE and then electrotransferred to nitrocellulose membranes (Bio-Rad, Hercules, CA, USA). Nonspecific binding was blocked with 5% fat-free milk for 1 h at room temperature. The membrane was then separately incubated with anti-TRF2 antibody (diluted 1:1000; Abcam, Cambridge, MA, USA), anti-Rap1 antibody (diluted 1:2000; Abcam), anti-ATM antibody (diluted 1:5000; Abcam), anti-phospho-ATM (S1981) antibody (diluted 1:5000; Abcam), anti-γH2AX antibody (diluted 1:1000; Upstate, Waltham, MA, USA), anti-phospho-p53(S15) antibody (diluted 1:1000; Cell Signaling, Danvers, MA, USA) or β-actin antibody (diluted 1:10 000; Abcam) overnight at 4 °C. After binding of the horseradish peroxidase-coupled secondary antibody (diluted 1:2000; Santa Cruz Biotechnology, Santa Cruz, CA, USA) at room temperature for 2 h, antigens were visualized by enhanced chemiluminescence (ECL-kit, Santa Cruz Biotechnology). All results were representative of three independent experiments.

### Real-time PCR

Total RNA was extracted using Trizol (Invitrogen, Carlsbad, CA, USA), and cDNA was synthesized using the PrimeScript RT reagent kit (TaKaRa Biotechnology, Dalian, China), according to the manufacturer's recommendations. A LightCycler FastStart DNA Master SYBR Green I System (Roche, Basel, Switzerland) was used for the real-time PCR. The 25-μl reaction contained 12.5 μl of SYBR Green qPCR master mix (TaKaRa Biotechnology, Dalian, China), 10 nmol of each primer, 2.0 μl of the DNA template and 8.5 μl of dH_2_O. The PCR cycling conditions consisted of 40 cycles at 95 °C for 30 s, annealing at 58 °C for 40 s and extension at 72 °C for 30 s. The mRNA level of β-actin was used as an internal control, and the reaction mix without template DNA was used as a negative control. All of the samples were measured three times independently, and the resulting fluorescence curves represented the number of copies expressed. The following primer sequences were used:

β-actin, 5′-ATAGCACAGCCTGGATAGCAACGTAC-3′ (forward)

and 5′- CAC CTTCTACAATGAGCTGCGTGTG-3′ (reverse);

and Rap1, 5′-GCCACCCGGGAGTTTGA-3′ (forward)

and 5′-GGGTGGATCATCATCACACATAGT-3′ (reverse);

and TRF2, 5′-CTGAGCTCACACCACTGGAA-3′(forward)

and 5′-GCATCTTCTGCTGGAAGGTC-3′ (reverse).

### Plasmid construction and transfection

Two pairs of hairpin siRNA template oligonucleotides for Rap1 based on pSilencer3.1-H1 vector (Ambion, Austin, TX, USA) were designed by siSearch. Oligo-1:

5′-GATCCGTGTAGCTCGGAGGATTGAATTCAAGAGATTCAATCCTCCGAGCTACA TTTTTTGGAAA-3′ and 5′-AGCTTTTCCAAAAAATGTAGCTCGGAGGATTGAATCTCTTGAATTCAATCCTCCGAGCTACACG-3′. Oligo-2: 5′-GATCCGTTGGATGTATTTACAGCTGTTCAAGAGACAGCTGTAAATACATCCAA TTTTTTGGAAA-3′ and 5′-AGCTTTTCCAAAAAATTGGATGTATTTACAGCTGTCTCTTGAACAGCTGTAAATACATCCAA CG-3′. These oligonucleotides were annealed and ligated into vector, respectively. According to the manufacturers' instructions, pSilencer3.1-Rap1 siRNA plasmids 1 or 2 were transiently transfected into SGC7901/VCR using LipofectamineTM 2000 (Invitrogen). pSilencer3.1-H1 alone was transfected as negative control. Further studies were taken at 72 h post transfection. Similarly, previously constructed pcDNA-TRF2^7^ and pSilencer3.1-Rap1 siRNA plasmids were cotransfected into SGC7901. pcDNA3.1 and pSilencer3.1-H1 were cotransfected as negative control.

### Drug sensitivity assay

Sensitivity of gastric cancer cells to etoposide was evaluated using MTT assay. Cells were seeded onto 96-well plates (1 × 10^4^ cells per well) and incubated for 24 h. Etoposide was added and incubated for 72 h. Then treated cells were incubated with 20 μl of MTT solution (5 mg/ml) in PBS for 4 h. After removing the medium, 150 μl of dimethylsulfoxide (Sigma) was added to each well to dissolve crystals. Absorbance at 490 nm was measured with a microplate reader BP800 (Biogit). Dose-effect curves of etoposide were drawn on semilogarithm coordinate paper and IC_50_ values were determined.

### Statistical analysis

Data values were expressed as means±s.d. Statistical significance was assessed by one-way analysis of variance and GLM repeated measures by using SPSS 21.0 statistical software (SPSS, Inc., Chicago, IL, USA). A value of *P*<0.05 was considered significant.
